# Unsupervised anomaly appraisal of cleft faces using a StyleGAN2-based model adaptation technique

**DOI:** 10.1371/journal.pone.0288228

**Published:** 2023-08-03

**Authors:** Abdullah Hayajneh, Mohammad Shaqfeh, Erchin Serpedin, Mitchell A. Stotland

**Affiliations:** 1 Electrical and Computer Engineering Department, Texas A&M University, College Station, TX, United States of America; 2 Electrical and Computer Engineering Program, Texas A&M University, Doha, Qatar; 3 Division of Plastic, Craniofacial and Hand Surgery, Sidra Medicine, and Weill Cornell Medical College, Doha, Qatar; Mae Fah Luang University, THAILAND

## Abstract

A novel machine learning framework that is able to consistently detect, localize, and measure the severity of human congenital cleft lip anomalies is introduced. The ultimate goal is to fill an important clinical void: to provide an objective and clinically feasible method of gauging baseline facial deformity and the change obtained through reconstructive surgical intervention. The proposed method first employs the StyleGAN2 generative adversarial network with model adaptation to produce a normalized transformation of 125 faces, and then uses a pixel-wise subtraction approach to assess the difference between all baseline images and their normalized counterparts (a proxy for severity of deformity). The pipeline of the proposed framework consists of the following steps: image preprocessing, face normalization, color transformation, heat-map generation, morphological erosion, and abnormality scoring. Heatmaps that finely discern anatomic anomalies visually corroborate the generated scores. The proposed framework is validated through computer simulations as well as by comparison of machine-generated versus human ratings of facial images. The anomaly scores yielded by the proposed computer model correlate closely with human ratings, with a calculated Pearson’s r score of 0.89. The proposed pixel-wise measurement technique is shown to more closely mirror human ratings of cleft faces than two other existing, state-of-the-art image quality metrics (Learned Perceptual Image Patch Similarity and Structural Similarity Index). The proposed model may represent a new standard for objective, automated, and real-time clinical measurement of faces affected by congenital cleft deformity.

## 1 Introduction

Cleft lip with or without associated cleft palate (CL +/- CP) is one of the most common major congenital anomalies. The National Birth Defects Prevention Network 2017 Congenital Malformations Surveillance Report, which studied the United States birth cohort between 2010–2014, reported CL +/- CP prevalence at 1 in 1000 live births [1]. Surgical management may involve a series of reconstructive interventions through the childhood and adolescence years, and sometimes beyond. Unfortunately, current state-of-the-art surgery cannot erase all vestiges of the facial deformity, thus leaving some affected patients with lifelong psychosocial burden [2, 3]. Cleft lip deformity manifests itself across a spectrum of severity from “incomplete unilateral” to “complete bilateral” depending on the extent of congenital separation of the central philtral region and the two adjacent lateral lip elements on either side. It would be highly beneficial for treating surgeons to have a universally available, objective, and user-friendly method to measure cleft lip severity. This would facilitate (i) impartial self-assessment of clinical outcome on the part of the surgeon, (ii) meaningful discussion with patient/parent, (iii) outcome comparison between different surgical techniques and surgeons, (iv) enhanced surgical planning and education, (v) research on factors associated with cleft severity, and (vi) explicit characterization of the clinical needs and benefits of surgery for third-party payers. While a number of different methods for facial measurement exist, most have been used for research purposes only, and suffer from some combination of being technique- or technology-dependent, susceptible to human bias, or simply infeasible for use in a real-time clinical setting [4–27].

An ideal facial anomaly detection and measurement system should demonstrate (i) fine sensitivity: an ability to discern the types of subtle facial irregularities that matter to patients, (ii) order-preservation: reliably ranking anomaly severity within sets of images with a hierarchy corresponding to human appraisal, (iii) indifference to extraneous variation: tolerance to variations in age, gender, race, pose, lighting, etc., and (iv) comprehensibility: revealing an interpretable process of distinguishing subtle zones of facial aberration, e.g., through the provision of corresponding heat detection maps.

Training an effective anomaly detector, if using a supervised training model, is necessarily challenged by the scarcity of anomalous data. Moreover, it is fair to assert that no facial norm (or population average) can be used as a standard against which all faces of different age, gender, ethnicity, and severity of deformity can be compared. Thus, we have previously introduced the concept of comparing any given facial deformity to its counterpart normal transformation obtained using the StyleGAN facial generator [28, 29]. Herein, we extend the prior work by developing a model that provides more realistic facial normalization (employing StyleGAN2 [30]) with better computational performance and stability, along with a more precise and streamlined system for anomaly detection and measurement using a pixel-wise subtraction method.

## 2 Related work

**Fig 1:**
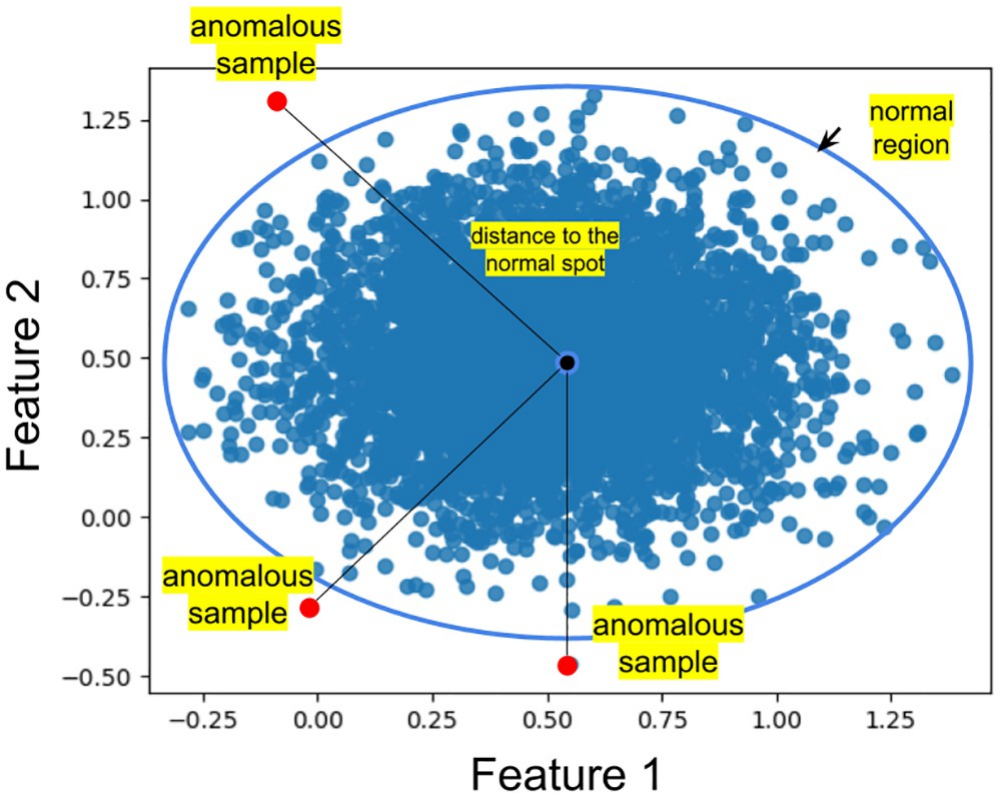
Example of a dataset of 1000 samples from Gaussian distribution with normal boundary arbitrarily established. Anomaly detection methods identify outliers as anomalies and calculate their distance from the center of the distribution.

Previous descriptions of anomaly detectors intended for medical application have targeted structural outliers in images including from CT scans [31] and radiographs [32] of the chest, and mammograms [33]. Those using machine learning methods for anomaly detection have depended on explicit modeling of the normal data distribution after first transforming it into a feature space. Newly introduced samples were then defined as anomalous if located outside the established boundaries of the normal distribution, as illustrated in Fig 1.

Erfani et al. used Support Vector Machines (SVMs) trained on one category of samples that represented features offered by a trained deep belief network model [34]. Using various types of non-medical, low dimensionality data from the UCI Machine Learning Repository [35], each individual dataset (< 600 features/set) was transformed into an alternative feature space where its contents could be better discriminated, allowing for clearer separation between normal and anomalous samples. However, it is unclear whether this type of SVM model would apply itself feasibly to higher dimensionality data such as 1024 × 1024 × 3 pixel images. A different approach employed a Random Forest classifier trained on a publicly available dataset consisting of 384 optical coherence tomography images to detect macular degeneration in an elderly population. Using their model to process 384 samples (115 normal images and 269 images with expert-confirmed macular degeneration) a 96% accuracy was reported [36]. Despite its success, this Random Forest-based method is a supervised learning approach which may not be applicable for the detection of rare anomalies within more complex images, such as those depicting the human face. Seeböck et al. used a variational autoencoder with a one-class SVM model to identify anomalous regions in a retinal imaging dataset with 86.6% accuracy [37]. This represented a good example of unsupervised anomaly detection, however this method employed a complex architecture which may not execute efficiently when processing high dimensionality images.

The emergence of Generative Adversarial Networks (GANs) offers an alternative approach to the design of anomaly detection and localization systems [38]. A GAN can be adapted to create a replica version of an anomaly-containing image—except with the anomalous portion corrected. This presents an opportunity for a more customized anomaly measurement by allowing for comparison of any raw image to its GAN-transformed counterpart, rather than to an arbitrarily determined population norm. In 2017, the AnoGAN framework introduced the application of this concept for the detection of anomalies within retinal tomograms [39]. The described model also demonstrated an ability to localize and severity-score anomalies within an image. AnoGAN was trained from scratch using one million patches to generate new and unique replicas of normal retinal tomographic images. An AnoGAN-generated image most similar to any raw retinal tomogram under consideration was obtained by finding an optimized latent vector corresponding to the required generated sample. The loss function employed for this optimization used both the generator and the discriminator of the GAN. Then, a heatmap was generated by defining the residual image as the pixel-wise difference between the generated image and the real one. The scoring system was built based on the loss function used during the latent mapping operation.

Boyaci et al. [29] reported on the first utilization of a GAN for the assessment of facial anomalies in which the original StyleGAN facial generator [28] was used to normalize faces depicting a variety of different deformities. The loss function pertaining to the optimization in their method employed two components focused on the similarity and averageness of an image. The similarity component attempted to preserve all normal features within an abnormal facial image, while averageness functioned to transform the face towards as close as possible to the “average face”. Following normalization of a raw facial image portraying deformity, a separate neural network trained using human ratings of normal and abnormal facial images was able to generate a “distance-from-normality score” for any raw image. The normalization procedure for this system was not stable in the sense that the convergence was inconsistent, thus requiring adoption of the average result of nine re-executed normalization operations as a final output. The computational overhead of this model requires up to 8 minutes to generate a score for an individual image using an ordinary GPU-based laptop.

The current method described here offers a number of important advantages over our previous work [29], including reliable convergence within an average time of 136 seconds. This is because only a single normalization operation is required due to the use of a tuned similarity loss function that allows for more optimization flexibility (see Methods section 3.2.1). The new system is now supported with a model adaptation step that provides more identity-preserving features of the original face that might not otherwise appear without this function. The instability issue of the previous model has now also been resolved by implementing StyleGAN2’s latent optimization method with acceptable normalization output. Finally, an important aspect of the new method is that the facial scoring pipeline employs simple image processing and comparison algorithms. This makes the proposed method more practical to execute on any ordinary computational device.

The main contributions of this paper are the following:

- a novel StyleGAN2-based model adaptation algorithm that incorporates additional identity-preserving facial features to fine-tune normalized cleft faces beyond what is achievable with the standard StyleGAN2 projection-based algorithm.
- a heat map, yielded by an image processing pipeline, to compare any input raw image with its normalized counterpart.
- a heat map-based facial scoring system to evaluate and sort samples according to their proximity to normality.

The rest of the paper is structured as follows. Section 3, Methods, briefly defines the mathematical notations required to model the facial anomaly scoring problem and presents the overall research methodology including image preprocessing, image normalization, color transformation, morphological erosion, heat map generation, and anomaly scoring steps are described in detail. Also, evaluation criteria, aspects pertaining to data collection and human ratings are presented. Section 4, Results, reports on the performance of the proposed method and compares it to existing metrics. Section 5, Discussion, summarizes the contributions of this work, outlines the advantages and limitations of the proposed pipeline. Section 6, Conclusion, proposes several future research directions to overcome the observed limitations.

## 3 Materials and methods

This study was approved by the Institutional Review Boards (IRBs) of Sidra Medicine and Texas A&M University. The proposed research model aims to measure the difference D between an abnormal facial image and its normalized counterpart to create a score that describes the severity of the input anomaly. For the purposes of this investigation, we obtained 61 facial images of pediatric patients with various types of cleft lip deformity from the clinical practice of the senior author M.A.S. (each with IRB-approved signed consent), as well as facial images of 64 normal children generated by StyleGAN2. The individuals in this manuscript have given written informed consent (as outlined in PLOS consent form) to publish these case details.

**Fig 2:**
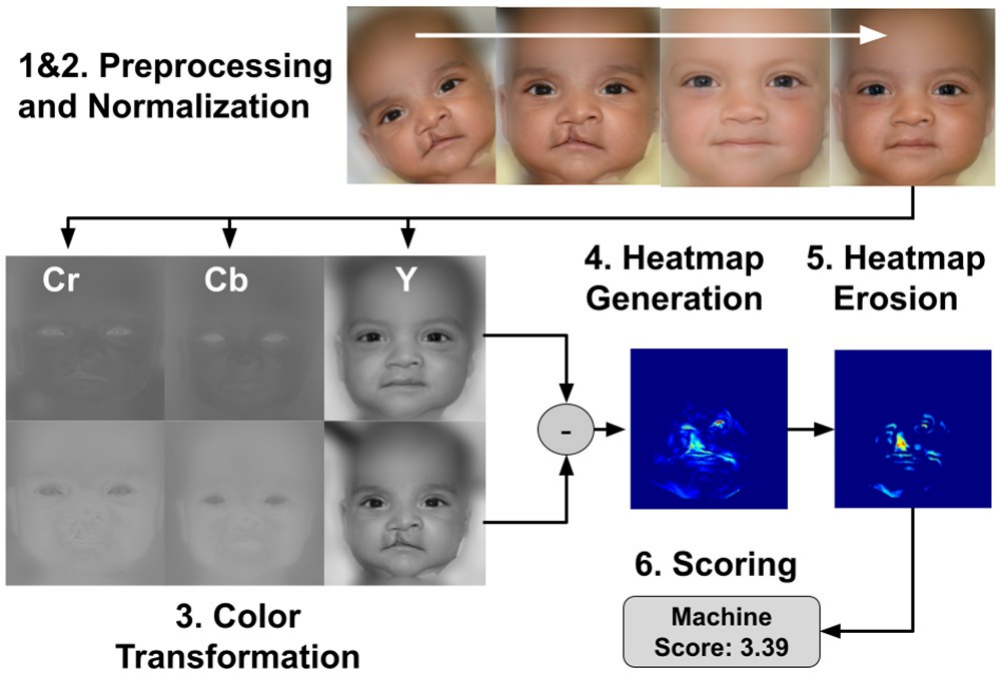
In order to develop an automated facial scoring system several steps were required: Image preprocessing, face normalization, color transformation, heat map calculation, morphological erosion and anomaly scoring.

The overall study methodology is summarized in Fig 2.

**Fig 3:**
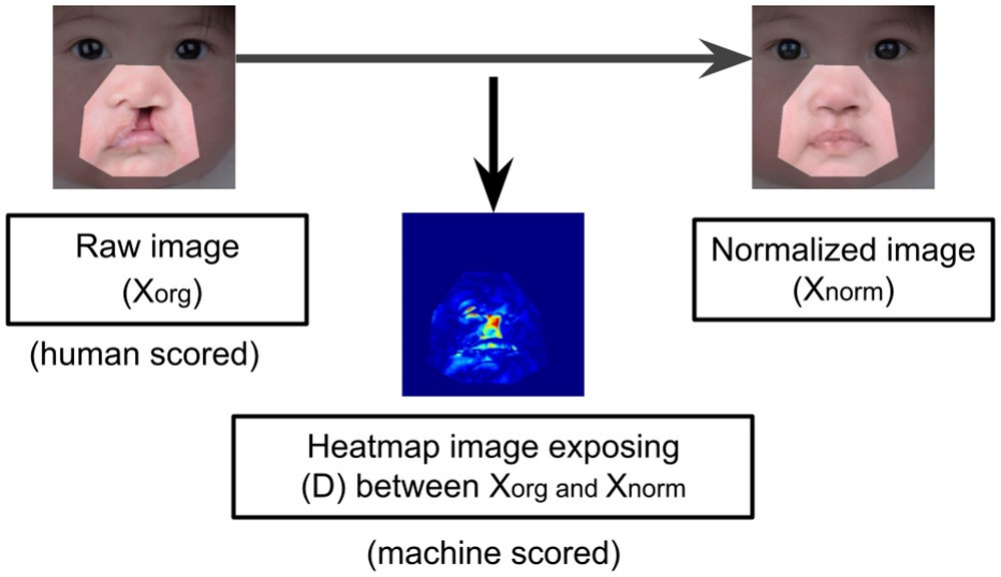
The objective of the proposed method is to obtain a machine score that aligns closely with a human rating by utilizing a difference map generated by comparison of original image with its normalized counterpart.

Let $x_{org}\in N^{n\times m\times c}$ denote the image containing the human face with cleft anomaly, with *n*, *m* and *c* representing its height and width and the number of color channels, respectively. The main goal is to assess the deviation of *x*_*org*_ from normality by calculating an index S∈R. Index *S* is obtained by building a relationship between a difference map $D\in R^{n\times m}$ between *x*_*org*_ and its normalized counterpart *x*_*norm*_, having the same dimensions as *x*_*org*_, and containing the same face but with the anomaly region suppressed and replaced with normal facial features (as illustrated in Fig 3).

### 3.1 Image preprocessing

In the first preprocessing step, the face inside the image *x*_*org*_ was detected and localized using the Haar classifier [40], which is a conventional face detection model. The area ratio of face-to-background needed to align with the FFHQ [28] dataset upon which the StyleGAN2 was trained (60% face to 40% background). If the ratio was small, an appropriate area of background was removed;if the ratio was large, the background was enlarged by blurring it and placing 8 horizontally/vertically flipped replicates of the image around it. This helped to match the shape of the input image with the StyleGAN2 generator input shape (1024 × 1024 pixels). To create a blurred background of the image around the face, a mask image is generated by separating the foreground from the background pixels of the original face image using the previously detected face. Afterwards, a Gauss smoothing filter [41] is applied to the mask to smooth the transition between the foreground and the background areas. Then the range of values of the mask image are converted from 0–255 to 0–1 and multiplied with the original face image.

**Fig 4:**
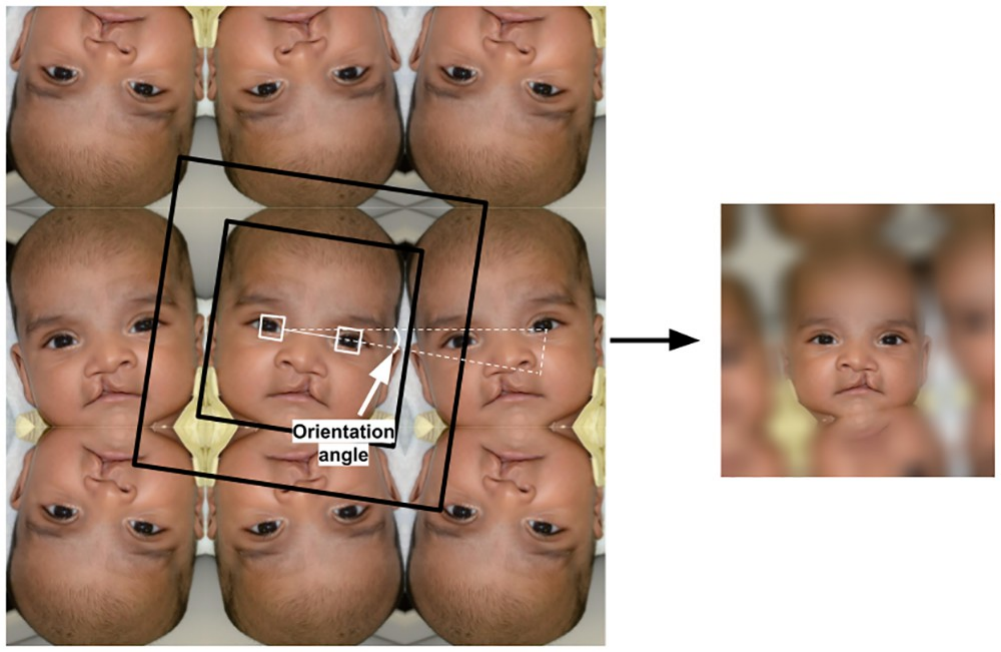
Applying different preprocessing steps to generate consistent images with the StyleGAN2 pretrained model. This includes adjusting the background, scale, and rotation/flip orientation.

The next preprocessing step is to detect and correct the orientation of the face inside the image. This is conducted by detecting the eyes in the face by using 68 landmarks of the face. The goal is to secure horizontal alignment of the eyes inside the image. Next, the distance between the eyes is measured and the whole image is scaled up or down so that the distance between the centers of the eyes is equal to 100 pixels, a condition which ensures consistency with the StyleGAN2 pretrained model. Finally, 1024 × 1024 pixels are cropped around the face location. Fig 4 illustrates the overall preprocessing steps.

### 3.2 Image normalization

**Fig 5:**
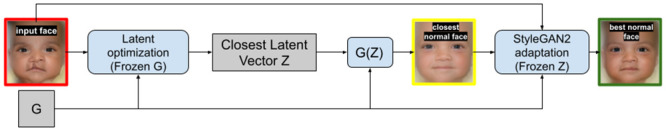
The proposed normalization algorithm searches for the latent vector most closely matching the input face. The latent vector is then frozen and the StyleGAN2 model is optimized to reconstruct more facial details without anomalies. G refers to the StyleGAN2 generator network.

After obtaining a well-aligned face image *x*_*org*_ through the preprocessing step, a normalized version *x*_*norm*_ of the original face was created by utilizing the StyleGAN2 face generator which produces unique and high quality faces. The overall proposed normalization algorithm is illustrated in Fig 5. It consists of two successive operations: Latent Optimization and StyleGAN2 Adaptation.

#### 3.2.1 StyleGAN2 latent optimization—Face inversion

For the sake of consistency in the overall pipeline, this step used the standard face inversion method proposed by the StyleGAN2 original paper, which is described in Algorithm 1. This algorithm optimizes the intermediate latent vector *W* as well as the 18 noise maps **n**_*i*_, *i* ∈ 0, 1, …, 17, present in the StyleGAN2 architecture to help in finding the best latent vector that encodes most of the facial features present in *x*_*org*_.

StyleGAN2 latent optimization starts by generating 10,000 random latent vectors *Z*, transforming them using the mapping network (see the S1 Appendix for more details about StyleGAN2 architecture) to produce 10,000 intermediate latent vectors *W* and averaging them to get an estimate of the mean intermediate latent vector *μ*. The algorithm optimizes *μ* to get an initial estimation of the latent vector corresponding to the closest generated face to the input image. The next step involves calculation of the similarity loss between the original image and the estimated image corresponding to the latent vector using the Learned Perceptual Image Patch Similarity (LPIPS) [42] distance measure $D_{LPIPS}$. The LPIPS loss function takes the following mathematical expression:

$$\begin{array}{c} D_{LPIPS}\left(x,y\right)=\sum_{l}\frac{1}{W_{l}H_{l}}\sum_{h,w}^{W_{l},H_{l}}\left|\right|w_{l}⊙\left({\hat{x}}_{hw}^{l}-{\hat{y}}_{hw}^{l}\right){\left|\right|}_{2}^{2}\;, \end{array}$$ (1)

where *l* stands for the layer index, *w*_*l*_, *W*_*l*_ and *H*_*l*_ denote the the learned weights, width and height of the layer *l*, respectively, and ⊙ represents the Hadamard product (vector element-wise multiplication).

To facilitate the latent vector search, the inversion algorithm additionally optimizes 18 randomly generated noise maps with different resolutions $n_{i}\in R^{r_{i}\times r_{i}}$, *r*_*i*_ ∈ {1024, 512, …, 8}, for face synthesis operation. As a consequence of optimizing the noise maps, some facial details may sneak into the noise maps during the synthesis operation. Therefore, a regularization term $L_{i,j}$ scaled by *α* is added to the overall loss, which depends on the original noise maps as well as scaled versions of noise maps **n**_*i*,*j*_, *j* > 0. These scaled versions are not part of the optimization. The $L_{i,j}$ regularization term measures the amount of randomness present in the noise maps and makes sure that the noise maps are still random.

**Algorithm 1** Face Inversion, Input: Face image *x*_*org*_ and StyleGAN2 generator model *G*. Output: Latent vector *w* and set of noise maps *n*_*i*_, *i* ∈ 0, 1, …, 17, *α* = 10^5^

**z** = {*z*_1_, …*z*_10000_} ← *U*{0, 1, …, 10000}

**w** = {*w*_1_, …, *w*_10000_} ← *G*(**z**)

$\mu \leftarrow \frac{\sum_{i}\left(w_{i}\right)}{N}$

$\left\{n_{1},n_{2},...n_{i}\right\}\leftarrow \left\{R^{r_{1}\times r_{1}},R^{r_{2}\times r_{2}},..,R^{r_{i}\times r_{i}}\right\}$

**while** not converge **do**

$L_{Image}=D_{LPIPS}\left(x_{org},G\left(\mu ,n_{i},..\right)\right)$

$n_{i,j}\leftarrow Downsample\left(n_{i},0.5\right),0\leq j<i,j\in N$

$L_{i,j}\leftarrow {\left(\frac{1}{r_{i,j}^{2}}\sum_{x,y}n_{i,j}\left(x,y\right)n_{i,j}\left(x-1,y\right)\right)}^{2}+{\left(\frac{1}{r_{i,j}^{2}}\sum_{x,y}n_{i,j}\left(x,y\right)n_{i,j}\left(x,y-1\right)\right)}^{2}$

$L\left(x,G\left(W,n_{i},..\right)\right)=L_{Image}+\alpha \sum_{i,j}L_{i,j}$

$\nabla w\leftarrow \frac{dL}{dw}$

 **while** *i* in (0, 1, …, 18) **do**

$\nabla n_{i}\leftarrow \frac{dL}{dn_{i}}$

  **n**_*i*_ = **n**_*i*_−∇**n**_*i*_

 **end while**

 *w* = *w* − ∇*w*

**end while**

**return** *w*, **n**_1_, **n**_2_, …**n**_*i*_

#### 3.2.2 StyleGAN2 pretrained model adaptation

The additional training of the StyleGAN2 model is called model adaptation, a step in which the generator internal weights are updated and forced to represent the remaining details of the input face and to improve the face image. A custom adaptation method is described in Algorithm 2. The core of this algorithm is to incrementally adjust the StyleGAN2 weights *W* and freeze the initial estimation of the latent vector *w* obtained from the previous step. The initial guess of the latent vector corresponds to the best normalized face *x*_*norm*_. This will add more identity-preserving details in the generated face except those related to the anomaly. This step continues for a proper number of training iterations and stops when most of the details are represented in the generated face. To consistently represent more details of the face, a carefully designed loss function was used during the adaptation step as shown in the following equation:

$$\begin{array}{c} L\left(x_{org},x_{norm}\right)=D_{LPIPS}\left(x_{org},x_{norm}\right)+D_{L2}\left(x_{org},x_{norm}\right). \end{array}$$ (2)

**Algorithm 2** Pretrained model adaptation. Input: Face image *x*_*org*_, its corresponding closest latent vector *w* and a StyleGAN2 generator *G*. Output: Adapted generator *G*′

*G*′ ← *G*

*W* ← *weights*(*G*′)

**while** not converge **do**

 *x*_*norm*_ ← *G*′(*z*)

$L\left(x_{org},x_{norm}\right)=L_{LPIPS}\left(x_{org},x_{norm}\right)+L_{L2}\left(x_{org},x_{norm}\right)$

$\nabla g\leftarrow \frac{dL}{dW}$

 *G*′ = *G*′ + ∇*g*

**end while**

**return** *G*′; *x*_*norm*_

**Fig 6:**
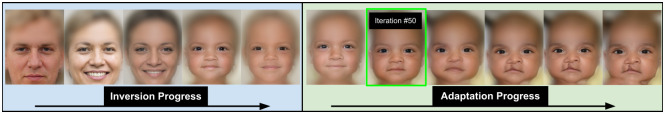
Facial transformation by applying the face inversion and model adaptation algorithms sequentially. The face in green border is the one that is taken as the best normalized version of *x*_*org*_ showing distinct features of the face without showing the anomaly. Having more adaptation iterations will reverse the normalization.

The above loss function seeks a compromise between reconstructing semantic details of the face captured by the Learned Perceptual Image Patch Similarity (LPIPS) loss $D_{LPIPS}$, and the details of image pixels present in both the original and generated faces indicated by $D_{L2}$. Both of these metrics are valid distance measures. This loss function is used to incrementally update the weights of StyleGAN2 to get the newly adapted generator *G*′ which contains the closest possible face *x*_*norm*_ to the original one *x*_*org*_. An example of the complete image normalization pipeline is described in Fig 6.

### 3.3 Color transformation

**Fig 7:**
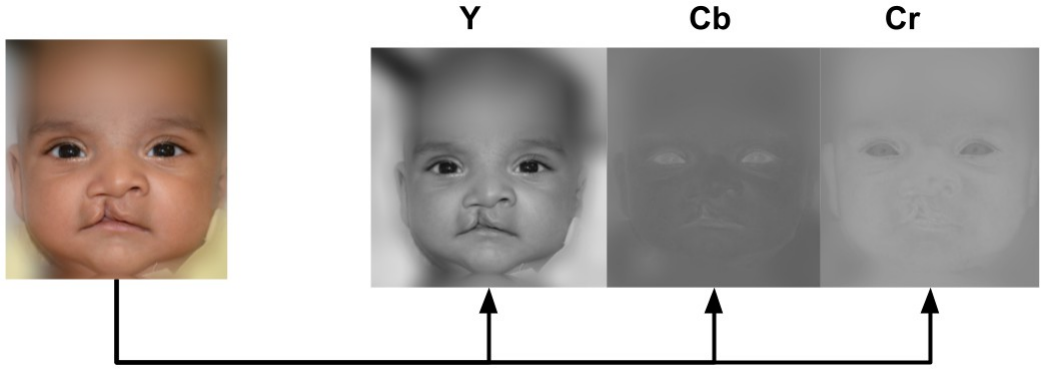
Transforming the facial image from RGB to YCbCr. Note that most of the variation is in the Y component. Cb and Cr exhibit negligible variation.

To ensure better discrimination between noise and face information, *x*_*org*_ and *x*_*norm*_ were transformed from Red-Green-Blue (RGB) color model to YCbCr (see Fig 7). Conversion from RGB to YCbCr color model (after gamma correction) were obtained using these transformations:

$$\begin{array}{cc} Y^{\prime } & =16+\left(65.481\cdot R^{\prime }+128.553\cdot G^{\prime }+24.966\cdot B^{\prime }\right)\\ C_{B} & =128+\left(-37.797\cdot R^{\prime }+74.203\cdot G^{\prime }+112.0\cdot B^{\prime }\right)\\ C_{R} & =128+(112.0\cdot R^{\prime }+93.786\cdot G^{\prime }+18.214\cdot B^{\prime }), \end{array}$$ (3)

where *R*′,*G*′ and *B*′ are the three color channels of the input image, *Y*′,*C*_*B*_ and *C*_*R*_ are the intensity, blue and the red differences [43], respectively.

The RGB color model describes a pixel information by the amount of each of the three colors included in it. The range of each color value is not linearly related to the intensity. Also, the RGB color system presents a lot of visual information redundancy in its three channels with no separation between color and intensity. YCbCr presents the advantage of separating the intensity values from chromaticity. YCbCr is widely used for skin color detection due to its ability to better represent color images with uneven illumination [44]. This helps in separating color from the illumination information.

### 3.4 Heatmap Generation via Pixel-wise Squared Error

The Pixelwise Subtraction Error (PSE) measures the difference in the color intensity between two images *x*_*org*_ and *x*_*norm*_ as follows:

$$\begin{array}{c} PSE\left(x_{org},x_{norm}\right)=\left(x_{org}-x_{norm}\right)⊙\left(x_{org}-x_{norm}\right), \end{array}$$ (4)

where ⊙ denotes the Hadamard product [45].

In our framework, we used the PSE similarity measure by considering the squared difference between each pixel in the original image and the corresponding pixel in the normalized version. The pixel-based distance measure included both the real anomalous difference and the difference that was caused by the change in illumination.

### 3.5 Morphological erosion for noise reduction

The normalized version of the face *x*_*norm*_ is indeed not an exact copy of the original *x*_*org*_ as it might include subtle changes in illumination (alluded to above) or in textural detail. These fine differences may or may not be detectable to human appraisal, but to address these artifacts we applied morphological erosion [46] on *x*_*org*_ as well as *x*_*norm*_ to reduce the effect that noise might have on our generated anomaly score.

The essence of the erosion process is to reduce objects boundaries and enlarge the size of holes. This is done by finding the minimum value of the neighborhood pixels at a specific location of the image. Let *F*(*j*, *k*) represent the pixel value of the grayscale image *F* under process at location (*j*, *k*). The erosion process over a 3 x 3 pixel neighborhood is implemented by means of this transformation:

$$\begin{array}{c} G(j,k)=MIN\{F(j,k),F(j,k+1),F(j-1,k+1),...,F(j+1,k+1)\}. \end{array}$$ (5)

This process is repeated around the image portion that is subject to analysis. We applied the erosion process on all the Y, Cb and Cr image channels as we assumed that the noise was contained in all the channels but with higher magnitude in the Y channel.

### 3.6 Anomaly score calculation and comparison

For the purposes of assigning cleft severity scores to all experimental images, we undertook both construction of a machine model as well as collection of human ratings for comparison. One hundred and forty-five human raters were recruited and were randomly shown one of ten image slideshows containing 15 cleft-affected children’s faces and 15 StyleGAN2-generated normal children’s faces. A total of 240 unique images were used. The order of the 30 images within each slideshow was randomly presented. The images were each displayed for only 3 seconds so as to discourage deliberation and yield more instinctive human responses. Subjects were requested to rate each image on a 1 (least normal) to 7 (most normal) scale. We report here only on images that received a minimum of 20 individual ratings (125 out of 240). The average number of human ratings per image under consideration was 25.

In order to generate the machine scores, the proposed framework was applied to the raw 125 samples under study. During the normalization step, 450 face inversion and 50 adaptation iterations have been performed to grossly obtain the desired facial details. The post-processed abnormality heatmap from the previous step was filtered to remove the unwanted parts of the image using a mask image *M*. Next, all the remaining pixels were summed up and divided by their total count *N*. This quotient represented an overall anomaly score indicating how much energy was contained in the heatmap. A higher quotient corresponded to a more severe facial anomaly. So as to enhance the sensitivity of the system to subtle facial anomalies, and to align the direction of severity of scoring with the human ratings, we then calculated the negative log transformation of the quotient, using the following equation:

$$\begin{array}{c} S=-log(\frac{\|M⊙\;PSE\left(x_{org},x_{norm}\right){\|}_{F}}{N}), \end{array}$$ (6)

where *x*_*org*_ and *x*_*norm*_ are the two face pairs. All scores derived from this calculation were then linearly scaled to facilitate comparable graph visualization. Note that for the evaluation phase, scores were similarly calculated for all 3 types of heatmaps under comparison (LPIPS, Structural Similarity Index Measure (SSIM) [47], and PSE) using the same operations.

The relationship between our machine-generated scores of the 125 facial images and corresponding human ratings of the same images was assessed using Pearson’s correlation:

$$\begin{array}{c} r=\frac{\sum \left(x_{i}-\bar{x}\right)\left(y_{i}-\bar{y}\right)}{\sqrt{\sum {\left(x_{i}-\bar{x}\right)}^{2}\sum {\left(y_{i}-\bar{y}\right)}^{2}}} \end{array}$$ (7)

where *x*_*i*_’s are the machine scores, *y*_*i*_’ represent the human scores, and $\bar{x}$ and $\bar{y}$ denote the means of the machine and the human scores, respectively.

### 3.7 Computing resources

We conducted the analysis on Python 3.6 using Pytorch, Opencv, Scipy and Skimage libraries on Intel i7–10751H CPU 2.6 GHz with Nvidia GeForce 2080 Super with Max-Q design.

## 4 Results

**Fig 8:**
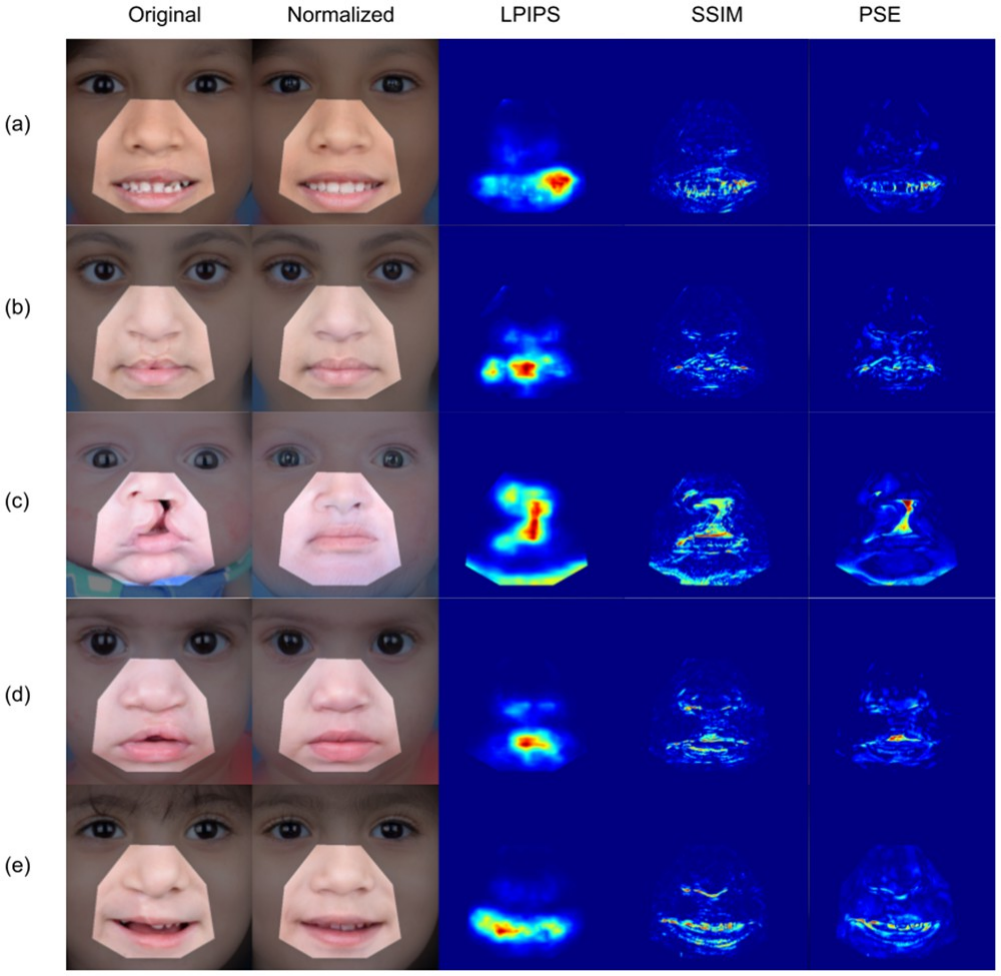
Comparison of various heatmaps generated using LPIPS, SSIM, and the proposed PSE method. The PSE approach demonstrates a more localized anomaly signal.

Representative examples of the proposed PSE and alternative heatmap generation methods are displayed in Fig 8. The original, normalized, and difference heatmap is shown for each cleft image. The oral and nasal areas are highlighted by masking. Abnormal regions under consideration are represented in the heatmaps by “hotter” pixels. Reviewing the three versions of heatmaps, the PSE method appears to light up anomalous cleft regions with more refined localization than the two peer techniques do.

Tables 1 and 2 show the Pearson correlation between machine and human scores for only the 61 real cleft faces and for the whole 125 experimental facial images, respectively. Each table shows the correlation coefficient with different combinations of image processing and heatmap generation methods. The correlation with and without the model adaptation step of image normalization is shown. Also, the correlations after masking everything except the oral/nasal region is compared with the correlations considering the entire face excluding eyes. Heatmaps and associated severity scores generated by the PSE machine-generated method showed an 88.7% and 82.8% correlation with human ratings for oral/nasal region and the entire face, respectively. The obtained correlations for PSE-based scores were achieved when applying both color transformation as well as the erosion operations to the PSE heatmaps. In case of the LPIPS-based scores, the color transformation step improves the correlation (79.4 to 83.5%) without applying the erosion step. Conversely, the SSIM-based scores show the worst performance and correlation degradation (from 81.5 to 68.6% in case of applying YCbCr transformation exclusively) and good improvement in case of adding the erosion operation only (81.5% to 84.9%). In general, the results show the superiority of the simple PSE among the LPIPS and SSIM-based scoring methods in almost all scenarios. Additionally, LPIPS works well with color transformation, while SSIM-based scores improves with the erosion operation.

**Table 1:** Pearson correlation coefficient between the human and machine scores of the 61 cleft faces under analysis, utilizing different combinations of heatmap generation and image processing approaches. The best combination is highlighted in bold below.

With Adaptation	Oral/Nasal Region Only				Entire Face (excluding eyes)			
Heatmap	Base	+YCbCr	+Erosion	+Both	Base	+YCbCr	+Erosion	+Both
LPIPS	79.4	83.5	79.2	83.1	66.4	77.4	66.4	77.2
PSE	85.2	86.1	87.4	88.7	81	81.9	81.5	82.8
SSIM	81.5	68.6	84.9	69.8	68	67.5	76.3	68.5
No Adaptation (StyleGAN2 [48])	Oral/Nasal Region Only				Entire Face (excluding eyes)			
LPIPS	60.7	63.6	61.1	59.5	47.2	61.8	47.7	56
PSE	68.8	69.6	70.1	69.9	62.7	64.5	64.4	65.7
SSIM	69	37.5	72.8	43	53.7	39	64.3	46.5

**Table 2:** Pearson correlation coefficient between the human and machine scores of the 125 StyleGAN2-generated and real cleft faces under analysis, utilizing different combinations of heatmap generation and image processing approaches. The best combinations is highlighted in bold numbers.

With Adaptation	Oral/Nasal Region Only				Entire Face (excluding eyes)			
Heatmap	Base	+YCbCr	+Erosion	+Both	Base	+YCbCr	+Erosion	+Both
LPIPS	0.84	88.8	84.2	87.9	77.8	86.2	77.5	85.1
PSE	89.9	89.9	88	90.4	87.1	87.5	84.7	87.1
SSIM	83.2	69.6	87.2	69	72.9	68.7	80.7	70.9
No Adaptation (StyleGAN2 [48])	Oral/Nasal Region Only				Entire Face (excluding eyes)			
LPIPS	79.1	81.2	79	80.1	71.8	79.6	71.8	79.2
PSE	80	79.4	75.4	75.8	75.9	75.7	71.4	71.4
SSIM	80.4	57.9	84.1	62.5	73.2	61.3	81	68.2

**Fig 9:**
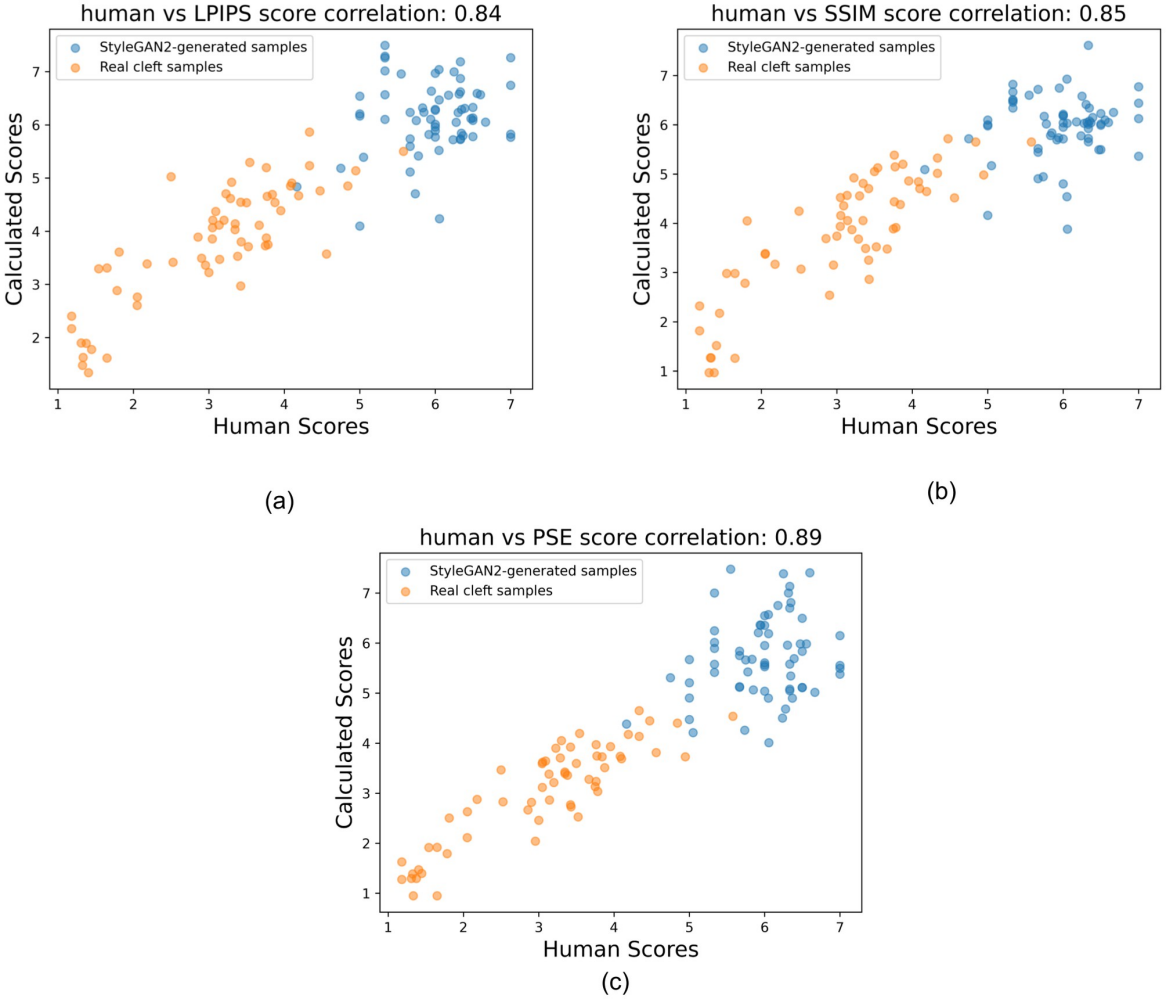
The Pearson correlation between human and machine scores of the 125 faces under study using different algorithmic approaches. The optimal heatmap and imaging processing combination for each of the 3 approaches is depicted here (as reflected in bold in Table 1) Note tighter alignment of human/machine scoring with the PSE method.

To visually demonstrate the effectiveness of the proposed scoring method compared to others, Fig 9 shows three plots of the human versus machine scores for the 125 faces under study. These plots corresponds to LPIPS, SSIM and PSE-based scoring methods, respectively. It is obvious that the PSE-based scores present better consistency than LPIPS and SSIM-based scores.

The average computation time required to evaluate the score of one sample, utilizing different combinations of heatmap generation and image processing approaches is shown in Table 3. An overhead of approximately 135 seconds is added for image normalization. The proposed method presents lower overall computational overhead relative to the approach in [29], which requires 9 minutes to score one image.

**Table 3:** Average evaluation time per sample in seconds for each combination of operations under analysis, when *x*_*org*_ and *x*_*norm*_ are provided. Another 135 seconds have to be added for each normalization step with adaptation to obtain *x*_*norm*_, while 123 seconds have to be added when using the standard StyleGAN2 normalization procedure.

Heatmap	Base	+YCbCr	+Erosion	+Both
LPIPS	1.297	1.757	1.672	1.769
PSE	0.096	0.096	0.104	0.104
SSIM	0.361	0.349	0.377	0.371

## 5 Discussion

The notable performance of the proposed model can be attributed to its multi-step methodology, each step contributing to its effectiveness. Image preprocessing was required to reconcile the input cleft images within the framework of the StyeGAN2 architecture. The normalization function reported here reprises our previously described innovation built off StyleGAN, but with important enhancements including (i) the use of a tuned similarity loss function available within the updated StyleGAN2 allowing for more optimization flexibility and resolving the prior instability issue, and (ii) a novel model adaptation technique that retains more identity-preserving features of the input image. Finally, the facial scoring procedure described here employs simple image processing and comparison algorithms, making the overall system more practical for use on any ordinary computational device.

Three key features of our method were (i) leveraging the capabilities offered by the StyleGAN2 facial generator [48] to generate high-quality normalized face images, (ii) complementing the anomaly score generated by the computer model with visual heatmaps to localize the facial abnormality and make the machine scores explainable, and (iii) incorporating conventional image processing techniques to improve the quality of the generated heatmaps and reduce the noise in the generated heatmap, which is needed in order to get more reliable machine scores of the anomalies. An important question that pertained to a given face image with cleft anomaly was: how can StyleGAN2 be utilized to find a matching normal face that does not show the cleft mouth anomaly and at the same time preserves the identity of the face? This was achieved by the *face inversion* operation which finds the vector in the latent representation of a pretrained StyleGAN2 face generator model *G* that transforms into a guess of the normal face image that most closely resembles the given abnormal face under evaluation. It should be noted that StyleGAN2 model was trained using normal face images only, and hence it is only capable of producing normal face images. The reason of naming the step of finding the latent vector as face inversion is that the process finds a latent vector given a face image instead of producing a face image given a latent vector, which is the primary common use of StyleGAN2.

The dimensionality of the StyleGAN2 latent vector is 512, and this number is a design parameter chosen by the creators of StyleGAN2. Thus, we stick to this number to ensure consistency of the proposed model with StyleGAN2 model. Having 512 dimensions of the latent vector is considered to be sufficiently good to compress the amount of information that distinguishes an individual face from other human faces. Also, choosing this number depended on the number of samples used to train the model to ensure the convergence of the learning process. Having higher dimensionality of the latent vector can help to achieve better normalization of the original images. However, this requires creating a new deep learning model with more training samples and more complex training process.

Different approaches were proposed in the literature to find the closest latent representation of a face in StyleGAN2. These approaches can be divided into two main categories. The first category is optimization based methods where a latent code is directly optimized for a fixed sample [30, 49–51], and the second category comprises encoder based methods where a separate encoder network is built and trained to predict the latent code for the sample [52–54]. Encoder based methods have the advantage of producing the corresponding latent vector in a single forward iteration through the encoder. This helps in significantly reducing the computation load overhead compared to the optimization based methods. On the other hand, optimization based methods can produce latent vectors associated with better quality images and closer normalization to the input image. This is important for our work since we need our method to be able to detect subtle changes in facial images. Additionally, a hybrid method lying between the two above mentioned categories was proposed in [55]. The hybrid method first finds an estimate of the latent code using a well trained encoder to shorten the latent optimization overhead. Then it employs an iterative optimization algorithm to refine the latent code and to better represent the semantics of the image in the latent space.

The chosen inversion method in our work resides in the category of optimization based methods and is considered as a standard inversion method. In general, this method iteratively calculates and minimizes a similarity loss between the input face image and a randomly generated face by adjusting a random latent vector using the back-propagation method [56]. The utilized loss function is based on the LPIPS loss $D_{LPIPS}$. LPIPS calculates the averaged difference between deep features extracted from two images using feature extractor networks such as pretrained VGG [57], Alexnet [58] or SqueezeNet [59].

After conducting the estimation of the normalized face during the inversion step, we observed that the obtained estimate still lacks some identity preserving details of the abnormal face. This may be due to the fact that the training dataset of StyleGAN2, which consists of 70,000 images of human faces, does not completely capture all variations among every individual human’s face. Another reason is that the face inversion step involves a highly nonlinear optimization problem and the numerical algorithm to solve it may fail to explore the entire latent space. This prompted for an enhanced normalization by applying additional training of the StyleGAN2 pretrained model in a Model Adaptation step. Several works have been proposed to adapt new datasets into a pretrained StyleGAN2 model [30, 60–62]. Most of them are designed to change the domain of the generated objects. Our approach benefits from these so that a small update can be applied to the generator to produce the required details of the original face. This small update does not alter the remaining latent space and its corresponding generated images. Also, we do not utilize the edited generator for any other task except to refine the target normalized face.

**Fig 10:**
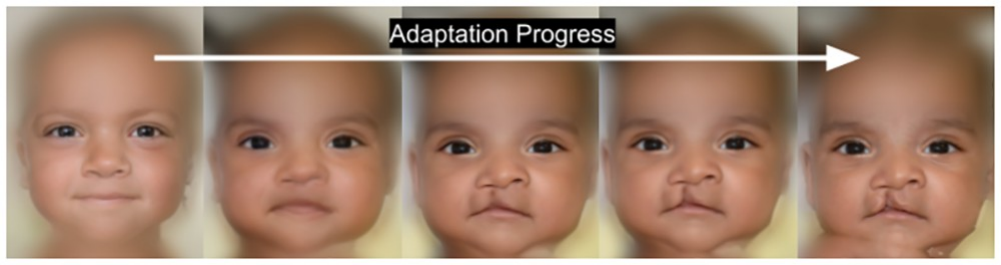
If we keep adapting the StyleGAN2 generator for more than 50 iterations, the model starts reconstructing the abnormal details of the original face. Here the cleft anomaly gradually appears in the generated face.

Selecting the number of adaptation iterations of the proposed algorithm depends on the amount of missing details we want to be present. If the input face is very close to normal, then less adaptation iterations are required, as the majority of the face details will be present in the first inversion step. A proper number of iterations to obtain most of the normal missing details of the generated face was found to be 50 adaptation iterations. If the adaptation proceeds with more iterations than this number, abnormal parts of the input face will be gradually reconstructed which harms the normalization result, as shown in Fig 10. Nevertheless, this observation can be useful for other applications, which are out of the scope of this work, such as the simulation of the recovery state of a cleft affected patient.

The bottom part of Table 3 shows the correlation between the machine predictions with the human scores, in the situation when the proposed StyleGAN2 model adaptation is not applied during the normalization phase. Clearly, the correlation is worse except for SSIM as a lot of facial details in the original image were not represented in the normalized face. This explains the importance of applying extra adaptation steps for the StyleGAN2 facial generator model.

One interesting idea to explore is to count the number of adaptation iterations that are needed to fully reconstruct the anomalous parts and to take that number as a measure of severity of the anomaly. The strong correlation between the number of iterations and degree of anomaly severity can be employed to generate an alternative severity index for facial deformities, a research direction that we plan addressing in a future companion paper.

One final comment on the normalization step is that it has a major limitation in its inaccuracy of reconstructing the eyes even with the state-of-the-art deep generative models. There could be different reasons for this difficulty including the fact that the eyes capture reflections of the surrounding environment in front of the person who is captured in the image. These reflections are difficult to reconstruct accurately by deep models including the StyleGAN2 generator. For the sake of our main purpose of scoring cleft abnormalities, this limitation can be mitigated by masking the eyes region.

Following the normalization step, heatmap generation was essential for obtaining the anomaly score. It was important for the heatmap to highlight the anomalous parts rather than the actual pixel difference. In general, almost all the similarity measures rely on heatmaps to calculate the similarity score. This makes it possible to test different methods in terms of their abilities to relate the heatmaps with consistent scoring systems. Generally, the heatmap can be created by comparing the original image with the normalized one by means of a similarity distance measure. Several similarity measures can be used to generate the heatmap. These measures can be divided into two main categories: shallow and deep measures, respectively. The first category uses an explicit definition of the difference between the two images.

Structural Similarity Index Measure (SSIM) is a popular shallow similarity measure that compares the difference of quality between two images in terms of color and contrast as well as structural differences. It also generates a heatmap depending on the above mentioned difference features. The SSIM between a window *x* any *y* of size *N* × *N* in *x*_*org*_ and *x*_*norm*_, respectively is expressed using the following equation:

$$\begin{array}{c} SSIM\left(x,y\right)=\frac{\left(2\mu_{x}\mu_{y}+c_{2}\right)\left(2\sigma_{xy}+c_{2}\right)}{\left(\mu_{x}^{2}+\mu_{y}^{2}+c_{1}\right)\left(\sigma_{x}^{2}+\sigma_{y}^{2}+c_{2}\right)}, \end{array}$$ (8)

where *μ*_*x*_, *μ*_*y*_ are the means of *x* and *y* respectively, *σ*_*xy*_ in the covariance of *x* and *y*, *σ*_*x*_ and *σ*_*y*_ are the variances of *x* and *y*, finally, *c*_1_ and *c*_2_ are two stabilization factors.

An example of a deep comparison method is LPIPS. Deep similarity measures have implicit definition of the difference between two compared images as they employ features extracted by deep neural networks pretrained on a dataset of different types of objects, i.e., they behave like objects feature extractors.

We found that, despite its simplicity, the PSE heatmaps are more intuitive and consistent with the generated scores than other types of heatmaps if supported with additional image processing techniques. On the other hand, heatmaps generated by LPIPS and SSIM do not greatly improve even if supported by additional image processing techniques. Another important feature of the PSE heatmap is the fact that it was able to highlight the structural changes as well as the large contiguous blobs of cleft abnormalities. Therefore, it turns out that the PSE heatmap is closer to the human intuition than the other generated heatmaps.

Color transformation step was useful to improve the heatmap generation step by producing an anomaly difference map between the abnormal face and the normalized one that highlights the abnormality location in the face but neglects other sorts of differences like small color changes due to lighting. For cleft abnormality, the anomaly causes mainly a geometrical (i.e., structural) change in the image rather than color change. The geometrical information of the image can be obtained more precisely by using the illumination intensity information than the color information. This required a representation of the image using a color system that separates the color information from the intensity. We found that representing the images *x*_*org*_ and *x*_*norm*_ in the YCbCr color system during the scoring process improves the correlation between human and machine ratings. People’s appraisals of facial abnormality are more sensitive to structural changes in the image rather than to color. Thus, we transform the image from the RGB into the YCbCr color space to better capture the black/white information. Interestingly, color transformation improves the correlation (79.4 to 83.5%) in case of LPIPS as well, unlike SSIM heatmaps. This is due to the fact that the highlighted anomaly regions were not dependent on the color information.

The abnormality score generated by the computer model is based on summing the energy in the generated heatmap of the difference between an input original face image and its normalized version. However, it should be noted that the generated heatmap will always have inevitable added noise on top of the main signal (i.e., the abnormality difference). The noise may come from two main sources. The first source is from the quality of the input source image that depends on the acquisition device (camera) that is used to capture the image. The other source is from the normalization step using StyleGAN2 generator that adds a specific level of structural artifacts when producing a face [63].

It is obvious that the noise in the generated heatmap is distributed all-over the face image, while the abnormality signal is concentrated in the oral and nasal region only. Therefore, in order to enhance the signal to noise ratio, we found that applying a mask to sum (and average) the energy of the heatmap within the oral/nasal region only gives better scoring of abnormality that is more closely correlated with human ratings.

Furthermore, the heatmap morphological erosion step was useful to reduce the noise that is due to the normalization step. Comparing the final correlation between human scores and the PSE-based scores with and without erosion confirms that this additional image post-processing is very useful and well-integrated with the PSE-based heatmaps.

Another important step for the abnormality scoring was taking the log transformation of the PSE-based scores. This was important to resemble the non-linearity nature of human appraisal of abnormalities. We claim that human judgment of facial deformities is very sensitive to small anomaly changes which gradually diminishes as the abnormality becomes larger and more obvious. In other words, human appraisal of abnormality is not linearly scaled with the abnormality area.

## 6 Conclusions

In this work, we proposed a method to normalize and then gauge the anomalous faces in a manner similar human judgment. A comparison between different combinations of face normalization, model adaptation, image processing and similarity measures have been tested. The proposed method demonstrated an improvement in terms of the computation speed and correlation with human scoring. Also, this study showed that localizing the abnormality regions in a patient’s face can be conducted using the power of Generative Adversarial Networks (GANs). Even if there is no representation in the GAN pretrained model, we were able to embed these details into the StyleGAN2 model using the classical backpropagation algorithm for some of the face details. This helped us to preserve the identity of the patient’s face for the generated image as well as to remove the abnormality regions and replace them with normal face attributes. Therefore, we were able to generate a heat map that highlighted the possible abnormal face parts using the pixel-wise difference between the original abnormal face and the normalized one.

It is worth to indicate that the severity index calculation method relies on pixel-wise subtraction, which measures the difference in intensity between the original face and the normalized counterpart. This means that some semantic image differences may not be detected, which may affect the overall scoring in some cases. Also, the evaluation was done using 125 human rated facial images. This number can be increased to confirm the performance improvements in more diverse cases. Also, a large number of fabricated cleft faces with varying levels of severity can be obtained by building a cleft face generator using the available real cleft faces. The number of adaptation steps is another parameter that can be optimized in the future work to generate a new anomaly index for facial deformities. Finally, different new deep learning models can be utilized in the future to improve the performance of the anomaly detection problem introduced in this work [64–70].

## Supporting information

S1 Appendix The StyleGAN2 generator architecture. This appendix provides a general overview about the StyleGAN2 architecture and its chosen design parameters.
(PDF)
