# Supplementary material for: Unsupervised anomaly appraisal of cleft faces using a StyleGAN2-based model adaptation technique
Source: PLoS One. 2023 Aug 3;18(8):e0288228. doi: 10.1371/journal.pone.0288228 (PMC10399833; doi:10.1371/journal.pone.0288228)
Supplement: S1 Appendix — This appendix provides a general overview about the StyleGAN2 architecture and its chosen design parameters. (PDF) [file pone.0288228.s001.pdf]

The StyleGAN2 Generator Architecture

Abdullah Hayajneh

June 27, 2023

The structure of the StyleGAN2 face generator G is depicted in Fig. S. 1. The input to the generator is a 512 dimensional latent vector z which encodes different features of the face. The StyleGAN2 model transforms the latent vector into a 1024×1024 dimensional face image by passing the latent vector through the mapping and synthesis networks. The aim of the mapping network is to obtain better features representation through an *intermediate* latent space $\mathcal{W}$, which is also transformed into *styles*. Moving in one direction in the latent space should generate a consistent facial appearance change in the corresponding image (e.g., skin color). During the face generation in the synthesis network, styles produced from the latent vector are used along with the convolutional layers to control the styles of the generated faces including aspects such as skin color, eyes size, mouth width, etc. In addition, random noise maps are employed to allow for more stochastic variations in the image domain.

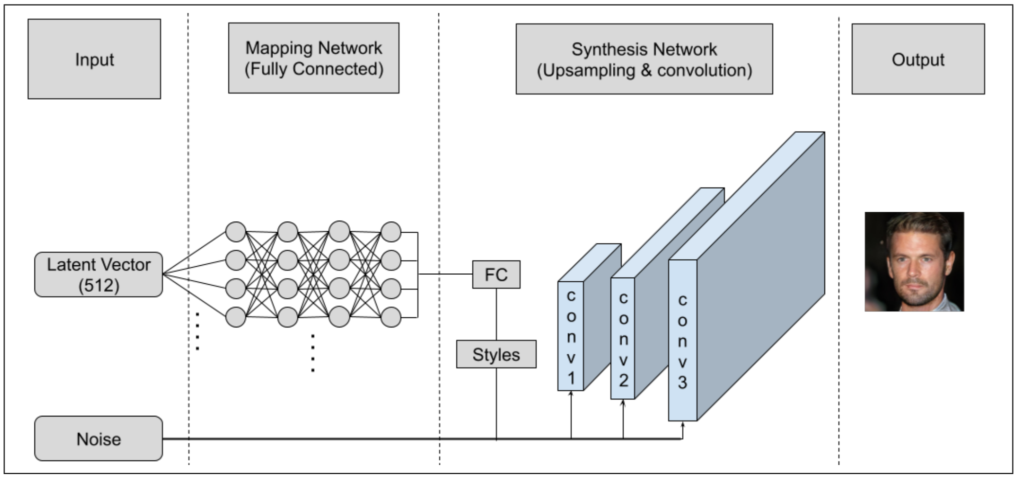

Fig. S. 1: General architecture of the StyleGAN2 generator. It consists of mapping and synthesis networks. The first network facilitates disentangled representation of the object features. The synthesis network produces diverse human faces by using the generated styles and noise maps with different sizes. At the pipeline output, this face generator can represent real-looking faces like the one shown at the right side of the figure [1].

References

[1] StyleGAN2 — Official TensorFlow Implementation;. https://github.com/NVlabs/stylegan2.
